# A Body Shape Index and Its Changes in Relation to All-Cause Mortality among the Chinese Elderly: A Retrospective Cohort Study

**DOI:** 10.3390/nu15132943

**Published:** 2023-06-28

**Authors:** Ning Yang, Jialu Zhuo, Suyi Xie, Zhihua Qu, Wei Li, Zixiang Li, Panpan Guo, Mingbo Gao, Huanlong Qin, Ting Han

**Affiliations:** 1 Department of Clinical Nutrition, Shanghai Tenth People’s Hospital, Tongji University of Medicine, 301 Yanchang Road, Shanghai 200072, China; elocin0922@163.com (N.Y.); lilyzhuo9302@sina.com (J.Z.); annaqu198997@163.com (Z.Q.); tenthnutrition@126.com (Z.L.);; 2 Shanghai Clinical Nutrition Quality Control Center, Shanghai Tenth People’s Hospital, Tongji University of Medicine, 301 Yanchang Road, Shanghai 200072, China; 3 Department of Medicine and Therapeutics, the Chinese University of Hong Kong, Prince of Wales Hospital, Hong Kong, China; suyixie@cuhk.edu.hk; 4 Laboratory for Heart Failure + Circulation Research, Li Ka Shing Institute of Health Sciences, Gerald Choa Cardiac Research Centre, Faculty of Medicine, the Chinese University of Hong Kong, Hong Kong, China

**Keywords:** A Body Shape Index, all-cause mortality, Chinese elderly, prospective study

## Abstract

Although recent evidence has revealed that a body shape index (ABSI) is correlated with the incidence of death among different ethnicities, there remains a paucity of studies investigating the impact of ABSI on mortality within the Chinese elderly. Our objective was to ascertain the link between ABSI, as well as its alterations over time, and all-cause mortality among Chinese aged 65 y and above. A total of 3789 participants were enrolled from the Chinese Longitudinal Healthy Longevity Survey (CLHLS). Cox regressions and restricted cubic splines were employed to assess the association of ABSI and relative changes with all-cause mortality. When nonlinearity was detected, a restricted cubic spline regression was subsequently conducted to compute hazard ratios and 95% confidence intervals. The median survival time was 46 months, and 1342 individuals (35.4%) were reported to have died. ABSI contributed independently to rising death rates among Chinese old populations according to univariate and multivariate Cox regressions. Statistically significant associations were also found stratified by age, sex, and lifestyle. A U-shaped association of ABSI changes with all-cause mortality (*p* = 0.027) was observed, indicating that old adults with stable ABSI during the follow-up period experienced the lowest risk of mortality. After multivariable adjustment, participants with a 10% reduction in ABSI changes had an increased 9.4% risk of death, while participants with a 10% rise in ABSI changes had an increased 1.9% risk. ABSI and its changes are predictors for all-cause mortality among the elderly Chinese population, which emphasizes the clinical importance of monitoring ABSI and keeping it stable over time.

## 1. Introduction

As the number and proportion of older individuals grows worldwide, promoting their health has become a pressing global issue [1]. Discovering predictors of mortality and accompanying factors in the aging population can pave the way for novel intervention tactics. Anthropometry contributes to older adults’ general mortality as a critical determinant, including body mass index (BMI) and waist circumference [2,3]. Also, the dynamic alterations observed in these anthropometric markers are intricately intertwined with mortality outcomes [4,5].

Body mass index (BMI) and waist circumference (WC) are frequently applied and simple indicators of body shape. However, these tools have some disadvantages. BMI cannot differentiate between fat and fat-free mass [6,7], while waist circumference is biased against individuals of different body sizes, making it less accurate [8].

To address these limitations, a newly invented anthropometric method named a body shape index (ABSI) was established by the Krakauer family in 2012 [9]. ABSI includes height, BMI, and WC in its calculation to provide a more comprehensive measure than single anthropometric parameters. High ABSI suggests that WC is greater than the anticipated value based on the person’s height and weight, which in turn highlights a central accumulation of body volume [9,10]. Previous studies have presented its validity in assessing fat mass, presenting its consistence with dual-energy X-ray absorptiometry body composition data [9]. ABSI surpasses the predictive ability of evaluating body shape of WC and BMI and has proven to be a vital risk factor for mortality and other diseases in some Western countries [11,12,13].

However, as the formula of ABSI was created according to the physiological characteristics of a Caucasian population, its efficiency in predicting the risk of mortality in the elderly Chinese population has not been clear. The obesity paradox among Chinese older adults that high BMI and WC protect against mortality always remains controversial [14,15]. Due to ABSI’s advantage of measuring body shape, it is necessary to conduct research into the connection between ABSI and mortality among the Chinese elderly. Additionally, past research has solely focused on the impact of baseline ABSI, measured at the initiation of the study, on future mortality, whereas the effects of ABSI changes at varying intervals on the risks of old adults’ mortality are unknown. Consequently, we employed data from the Chinese Longitudinal Healthy Longevity Survey (CLHLS) during 2011–2018 to explore the effect of ABSI, including its alterations, on the risk of death from all causes.

## 2. Materials and Methods

### 2.1. Study Design and Setting

The Chinese Longitudinal Healthy Longevity Survey (CLHLS) is a survey that invites older participants in various regions of China and prospectively follows them in the long run to examine different health outcomes [16,17]. Commencing in 1998, the survey conducts assessments periodically, with intervals of 2–3 years between each subsequent examination. Home-visit interviews for individuals aged 65 and above, including a substantial cohort of centenarians, are conducted by qualified interviewers using a structured questionnaire. For those who are dead or otherwise unable to respond, a spouse or a family member is solicited for replies to the survey questions. 

To capture the most up-to-date ABSI and comprehensively assess all-cause mortality among elderly individuals in China, our cohort study employed data spanning from 2011 to 2018 and determined 2014 cohort as baseline (*n* = 6066). The inclusion of this time frame was due to there being less influence by the social isolation imposed during the COVID-19 pandemic, which led to variations in the data collection methods employed after 2020, thus creating inconsistencies between the 2018 dataset and the subsequent data. In total, 3789 patients were recruited. Participants were excluded if the anthropometric results (*n*= 938) or basic demographic data (*n* = 282) were absent or lost in the follow-up in 2018 (*n* = 1057). The CLHLS study received ethical approval from the Research Ethics Committee of Peking University (IRB00001052e13074), and written informed consent was obtained from all participants.

### 2.2. Main Exposure and Outcome

Krakauer et al. developed ABSI in 2012, which served as the core exposure in our investigation [9]. ABSI was created by taking into account WC, height, and BMI, presenting a comprehensive indicator of both overall body shape and abnormal obesity. The ABSI values in this study were evaluated as continuous variables and categorized into quartiles for analysis (T1 ≤ 0.080; T2 0.081~0.088; T3 ≥ 0.089).

$$\mathrm{ABSI}=\mathrm{wc}/\left({\mathrm{height}}^{1/2}\times {\mathrm{BMI}}^{2/3}\right)$$

Standard anthropometric measurement techniques were applied, such as digital scales, or stadiometers. During measurements, participants were instructed to assume an upright position while relaxing and maintaining gentle breathing. A flexible tape measure was employed to measure the waist circumference at the midpoint between the lower margin of the ribcage and the highest point of the hip bone. This measurement was conducted twice, and the average value was recorded for the purposes of this survey. Weight and height were measured by qualified professionals and the values were precise to 1 cm and 1 kg. Body weight was measured when individuals were wearing light clothing. BMI was calculated by height (m)/weight (kg)^2^. 

For participants who could not stand, such as those who were bedridden, the CLHLS used alternative measurement methods. Weight might be measured using a bed scale or a chair scale. Height might be estimated using alternative methods, such as measuring the distance from the floor to the highest point on their head while lying down or using knee height measurements to calculate height. Waist circumference was measured using a flexible tape measure, which was placed horizontally around the smallest part of the participant’s waist, usually just above the belly button. All measurements were taken by qualified professionals by means of standardized protocols to ensure consistency and accuracy across the study population. 

In our study, we also examined the relative changes in ABSI over a four-year period as an additional exposure variable. This was motivated by previous evidence suggesting the potential of altered dynamics in conventional anthropometric measurements as predictors of illness and mortality outcomes. ABSI relative changes in 4 years were calculated using (ABSI in 2014 − ABSI in 2011)/ABSI in 2011 × 100%. The values were initially categorized into 3 groups: relative change −5% to 5%; relative change >5%; relative change <−5%, based on a similar study [5]. 

All-cause death status and death date from the baseline to 2018 were extracted from CLHLS database for analyzing the effect of ABSI and its changes over an extended period on the risk of all-cause mortality.

### 2.3. Covariates

To mitigate potential confounding effects, our analysis incorporated adjustments for demographic and sociological characteristics, lifestyle factors, and disease status. The sociodemographic features included age and gender, as well as marital status and residence. Lifestyle factors considered were whether participants currently smoked, drank, and performed physical exercise. Disease status was assessed based on self-reported common chronic diseases such as hypertension, diabetes, heart disease, and cardiovascular disease.

### 2.4. Statistical Analysis

To summarize the baseline characteristics of the study population by tertiles of ABSI, descriptive analysis such as mean ± SD and number and percentages were used where appropriate. One-way ANOVA was used to compare continuous variables among different ABSI tertiles. Proportion differences in categorical variables were compared using the chi-square test. Pearson’s correlation was applied to determine correlations between normally distributed variables, while Spearman’s correlation analysis was used for skewed distribution variables.

Survival curves were estimated according to the Kaplan–Meier method and compared using the log-rank test. Restricted cubic splines were applied to assess the linearity of the association. Cox regression analysis was used to evaluate the role of ABSI as an independent predictor of mortality by crude and adjusted models using the rest of the variables described above. We also performed analysis where risk was independently estimated for each tertile of continuous variables, compared with the highest group of ABSIs, in order to quantify the association between continuous variables and mortality.

Other Kaplan–Meier survival curves were generated for the study population classified according to the ABSI changes and a log-rank test was performed. Three major groups made up the whole research population: “Stability (−5% to 5%), Reduction (≤−5%), and Increase (≥5%)”. Then, due to the significant difference we explored, restricted cubic splines with 3 knots with 25th, 50th, and 75th percentiles were next applied for investigating the effect of relative ABSI changes on risk of death. A restricted cubic spline regression model was fitted where nonlinearity was evident.

Furthermore, we investigated if there were any variations in the association between the changes in ABSI and mortality when accounting for calf circumference. Calf circumference is a crucial indicator for malnutrition and sarcopenia, especially for the elderly [18]. We selected the cut-off points recommended by AWGS 2019 (Asian Working Group for Sarcopenia Consensus, 2019), which were calf circumference <34 cm in males or <33 cm in females [19]. Once there was indication of nonlinear interaction observed, we performed distinct analyses distinguished by calf circumference.

We also conducted sensitivity analyses to assess the robustness of our results applying to the participants without hypertension. All analysis was processed using R statistic software (version 4.0.3) and SPSS (version 26), with *p* < 0.05 as a statistically significant result. 

## 3. Results

**Figure 1:**
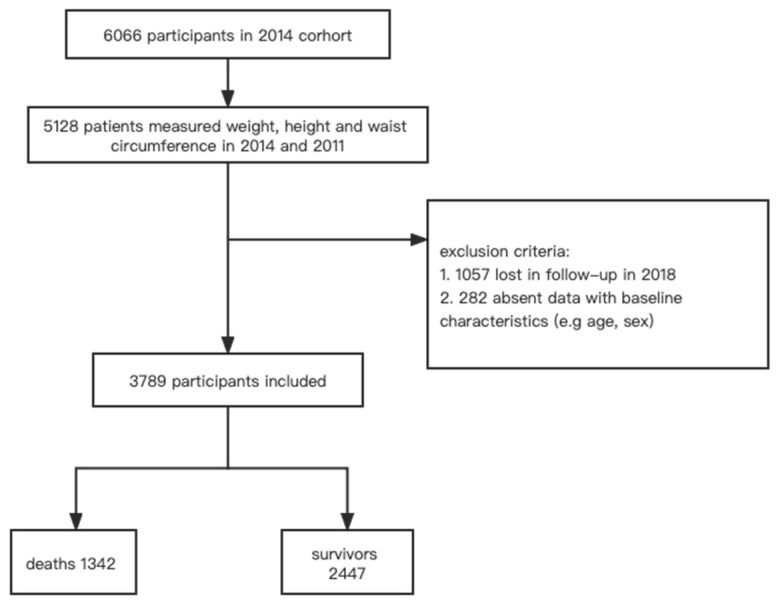
Study design flow chart.

Of the 6066 participants, 3789 participants with complete anthropometric and demographic data, as well as a record of lifestyle and certain chronic diseases, were selected from the 2014 cohort of CLHLS (Figure 1). Participants whose anthropometric or demographic data were incomplete (*n* = 938 + 282), as well as those who were lost in the follow-up in 2018 (*n* = 1057), were excluded.

### 3.1. Baseline Characteristics of Participants According to Tertiles of A Body Shape Index

Table 1 presents the demographic characteristics of the chosen study participants, categorized by ABSI tertiles at the baseline, who were subsequently monitored in 2018. Among them, the median complete survival time of the participants was 46 months, and 1342 individuals (35.4%) were reported dead at the 4-year follow-up time.

As determined by the baseline ABSI groups that the participants were divided into, 1262 participants were in the first tertile (T1), 1264 in the second tertile (T2), and 1263 in the third tertile (T3). Across the tertiles, 449, 385, and 508 deaths occurred in T1, T2, and T3, respectively. The mortality rates were 11.9%, 10.2%, and 13.4% in T1, T2, and T3, respectively (*p* < 0.001) (Table 1). 

The mean ABSI was (0.083 ± 0.01) m^11/6^kg^−2/3^ and the relative ABSI change during 2011–2014 was (−0.74 ± 15.98) %. Other biomarker mean values were (30.81 ± 6.49) cm for calf circumference, (80.98 ± 11.88) cm for waist circumference, and (21.81 ± 3.71) kg/m^2^ for body mass index. Rank correlation coefficients between ABSI and waist circumference and BMI were 0.52 and −0.264 (*p* < 0.05), respectively. Compared with participants in T1 and T2, those in the highest ABSI group (T3) were more likely to be women who lived alone, lacked physical exercise, and suffered heart diseases.

### 3.2. Association of Baseline ABSI with All-Cause Mortality

**Figure 2:**
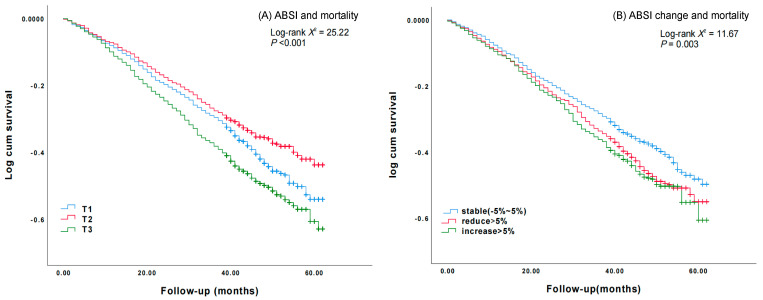
Kaplan–Meier curves in comparison of mortality risks by stratifying ABSI groups (**A**) and ABSI changes over time (**B**).

Of 3789 individuals, 1342 (35.4%) were reported dead at the 4-year follow-up time. Kaplan–Meier survival analysis indicated that the survival rates of participants among the tertiles of ABSI were statistically significant (*p* < 0.001). Participants in T3 had a higher risk of all-cause mortality in their following life than participants in either of the other tertiles (Figure 2). 

**Figure 3:**
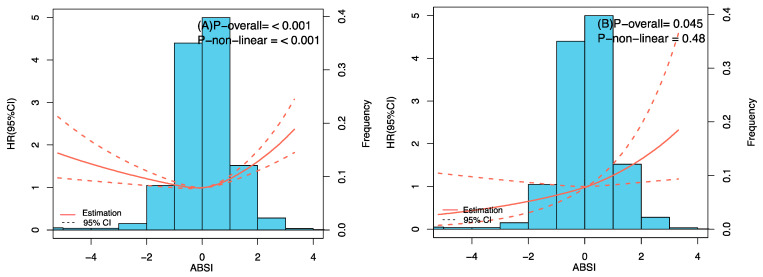
Restricted cubic spline curve between baseline ABSI and all-cause mortality. (**A**) Crude model. (**B**) Adjusted by sex, age, BMI, waist circumference, smoking, drinking, exercise, marital status, residence, hypertension, diabetes, and stroke or cardiovascular disease.

As shown in Figure 3, ABSI with full adjustment was associated with all-cause mortality in a linear positive relationship. The findings from both unadjusted and adjusted Cox proportional hazard models are presented in Table 2. Applying ABSI in the calculation of z-score as a continuous variable, the crude Cox regression analysis revealed hazard ratios (HRs) of 1.071 (95% CI: 1.015–1.131, *p* = 0.013), indicating a significant association between ABSI and mortality risk. This statistically significant association persisted in Model 3, which was additionally modified for potential confounders including demographic, medical history, and habits of drinking, smoking, and exercise, exhibiting a 29.3% higher risk of death from all causes with a SD ABSI z-score increase (HR: 1.293; 95% CI: 1.046–1.599, *p* = 0.018). Participants in ABSI T2 showed a 28.5% reduction in risk of death compared with T3 without adjustment for potential confounders. When additionally adjusted for confounders (Models 2 and 3), no significant association between ABSI tertiles and all-cause mortality was detected (Table 2). 

**Figure 4:**
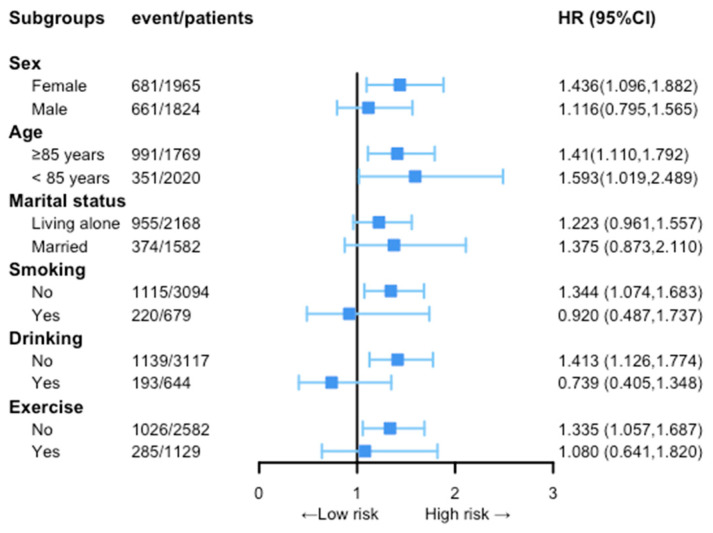
Stratified analysis of associations of ABSI with mortality. All models are fully adjusted by age, sex, BMI, waist circumference, marital status, residence, smoking, drinking, exercise, hypertension, diabetes, heart disease, and stroke or CVD. Abbreviations: HR, hazard ratio; 95% CI, 95% confidence intervals.

In subgroup analyses with sex, a significant increase in the risk of overall death was observed in females (HR: 1.436, 95% CI: 1.096–1.882, *p* = 0.009), whereas no significant association was observed in males. Categorized by age, there existed a positive correlation concerning ABSI and mortality. The results showed a higher mortality risk among participants <85 y than those 85 y (HR: 1.593 vs. 1.410, *p* < 0.05) while ABSI increased. However, an elevated ABSI was only significantly correlated with an increased risk of death among people who were nonsmokers (HR: 1.344, 95% CI: 1.074–1.683, *p* = 0.010), nondrinkers (HR: 1.413, 95% CI: 1.126–1.774, *p* = 0.003), and did not exercise often (HR: 1.335, 95% CI: 1.057–1.687, *p* = 0.015) (Figure 4).

### 3.3. Association of ABSI Changes (from 2011 to 2014) with Mortality from All Causes

The relative ABSI change in 4 years was (−0.74 ± 15.98) %. Among the participants, the ABSIs of 1246 individuals (32.9%) were relatively stable (−5~5%), those of 1142 individuals (30.1%) increased by more than 5%, and those of 1401 individuals (37.0%) were reduced by more than 5%. 

**Figure 5:**
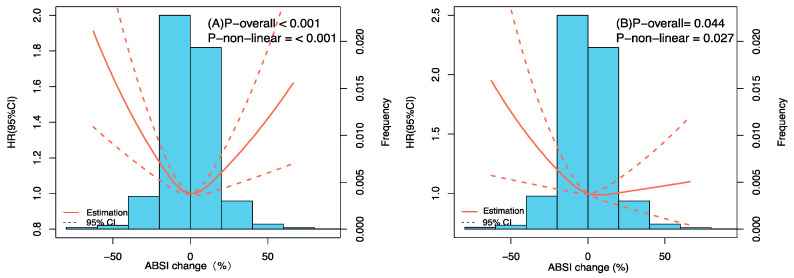
Restricted cubic spline curve between relative ABSI changes and all-cause mortality. (**A**) Crude model. (**B**) Adjusted by age, sex, marital status, residence, smoking, drinking, and physical exercise, hypertension, diabetes, heart disease, stroke and cardiovascular disease, calf circumference, BMI, waist circumference, and ABSI at baseline.

Kaplan–Meier survival analyses were statistically significant across the ABSI change groups (*p* = 0.003) (Figure 2). ABSI changes and overall mortality between the crude model and fully adjusted model were both shown to be correlated in a U-shaped pattern (*p* for nonlinearity = 0.027). The results indicated that the lowest probability of mortality was reached at a steady ABSI, and the probability rose after that, as demonstrated by the plot (Figure 5). For individuals who had a reduced ABSI (ABSI change percentage < 0), the hazard ratio of all-cause mortality per 10% drop in ABSI changes was 1.096 (95% CI: 1.091–1.100). In the ABSI change percentage > 0 group, the hazard ratio per 10% rise in ABSI changes was 1.062 (95% CI: 1.059–1.065). After multivariable adjustment, compared with those with stable ABSI changes, participants with a 10% reduction in ABSI changes had an increased 9.4% risk of experiencing death, while participants with a 10% increase in ABSI changes had an increased 1.9% risk of mortality (Table 3).

### 3.4. Subgroup Group Analysis of the Association of Relative ABSI Changes with All-Cause Mortality

**Figure 6:**
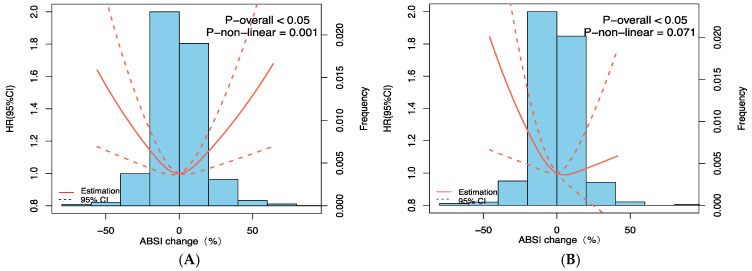
Restricted cubic spline curve between relative ABSI changes and all-cause mortality stratified by calf circumference. (**A**) Participants with ≥33 cm in females and ≥34 cm in males. (**B**) Participants with calf circumference <33 cm in females and <34 cm in males.

A statistically significant U-shaped association between changes in ABSI relative to the baseline and overall mortality persisted in individuals with calf circumference ≥33 cm in females and ≥34 cm in males (Figure 6). After adjustment for confounding factors, the hazard ratio per 10% reduction in ABSI over time was 1.062 (95% CI: 1.060–1.065) for values below 0, whereas above 0, the value per 10% increase was 1.015 (95% CI: 1.014–1.016). However, in individuals with calf circumference <33 in females and <34 in males, the U-shaped association was not evident; instead, an L-shaped association appeared (Figure 6). The hazard ratio per 10% reduction in ABSI over time was 1.088 (95% CI: 1.083–1.093) for values below 0, whereas above 0, the value per 10% increase was 1.015 (95% CI: 1.002–1.023).

### 3.5. Sensitivity Analysis

**Figure 7:**
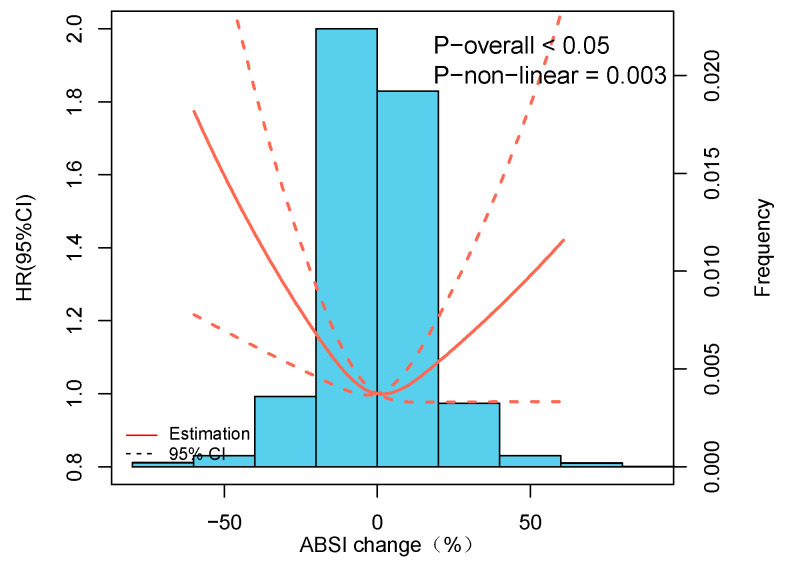
Association of ABSI changes with all-cause mortality in participants without hypertension.

These studies found a significant difference in participants with hypertension in ABSI tertiles. Therefore, we excluded individuals with hypertension to identify the association as well. A robust U-shaped relationship between ABSI alternations during follow-up and mortality from all causes still existed (Figure 7), consistent with our main results.

## 4. Discussion

By analyzing data from the 2014–2018 cohort in the Chinese Longitudinal Healthy Longevity Survey (CLHLS), this study’s findings suggest that ABSI is a significant and easily accessible predictor of all-cause mortality. The survival analysis using the Kaplan–Meier curve and Cox regression models further demonstrates that individuals with higher ABSI have significantly decreased survival outcomes. Going a step further, our investigation extended to identify a U-shaped relationship between ABSI changes and total cause of death. While accounting for baseline ABSI and other potentially confounding factors, we found evidence suggestive of a beneficial impact of ABSI stability on survival outcomes. 

Previous studies have established a robust association between older adults’ nutritional and functional status and their survival outcomes [20,21]. As a vigorous anthropometric measurement, BMI has been frequently utilized as an essential metric to assess the nutritional status of elderly individuals and offer valuable insights into their health [4,20,22]. However, BMI has deficiencies in capturing complex features such as fat distribution [23], thus being less effective in identifying the risk of central obesity, a known precursor of diseases [24]. WC has emerged as another significant compensation for BMI’s shortcomings [25]. Based on that, Krakauer NY et al. have introduced a new anthropometric technique known as ABSI, which assesses central obesity or overall adiposity in relation to BMI and waist circumference [9]. It provides an innovative approach to overcoming the limitations of traditional metrics. ABSI has demonstrated its significant accuracy and sensitivity for predicting the onset of health conditions as well, such as sarcopenia in overweight/obese individuals [26]; chronic kidney disease in older adults [27]; blood pressure variance in obese adolescents [28]; and prediabetes in Qatar adults [29]. In addition, numerous studies have also been performed to evaluate the effect of ABSI on health concerns, such as diabetes, cardiovascular disease events, and surgical complications [30,31,32]. Thus, ABSI acts significantly as a potentially valuable and reliable tool for assessing populations at risk.

However, research about the relationship between ABSI and mortality is inconclusive. Sun et al. conducted a study on diabetic US adults and found a positive association between ABSI and total mortality risk [11], whereas a Brazilian study found ABSI to have weak predictive capacity for mortality [33]. Such inconsistencies could be attributed to ABSI’s distinct characteristics across different ethnicities and populations. In our study, we discovered a linear positive association between ABSI and the risks of death in the multivariate Cox regression analysis. ABSI was deemed an independent predictor for mortality. While our results differ from those of another Chinese study with negative outcomes [34], speculations on the contradiction were made as follows. Firstly, the statistical precision of the original findings may have been constrained by a limited sample size of only 26 deaths, whereas our larger number of deaths (N = 1342) allowed for greater statistical power. Secondly, the previous study only investigated middle-aged Chinese men, where we focused on older individuals. Since the distribution of body fat and measures of obesity have been observed to fluctuate with age, it is crucial to consider age as a factor in analyses. Age may be a prognostic factor of ABSI in the Cox regression analysis results. In our analysis subgroup by age, individuals with high ABSI had a significantly higher risk of mortality than individuals with lower ABSI in either the old (<85 years) or super-old age groups (≥85 years). The hazard ratio among those who were younger than 85 years was remarkably higher than those who exceeded 85 years, which indicated that in the elderly, especially centenarians, it can be masked by other life-threatening illnesses, like senility, consistent with a previous study [11].

In addition, our study established an interaction between sex and ABSI for mortality risk, with a reverse outcome as well. We found that higher ABSI was significantly associated with increased risk of mortality in aged women but not in men, with the hazard ratio being remarkably higher in women than in men (HRs: 1.436 and 1.116). This discrepancy may be attributed to the characteristics of adipose tissue in Chinese individuals varying by sex, with older Chinese women having a higher rate of central obesity than men [35]. As obesity progresses, Asians, particularly postmenopausal women who undergo a reduction in estrogen levels, may deplete the storage capacity of their superficial subcutaneous adipose tissue compartment before others do [36]. Our results imply that ABSI may be a salient and practical predictor for Chinese elderly women to monitor in daily life.

Dynamic changes in various anthropometric measurements such as weight, BMI, and waist circumference in relation to mortality have been substantially proved [37]. Given the lack of evidence regarding associations of ABSI fluctuations with mortality, our study is the first to investigate the influence of ABSI changes on long-term survival in the Chinese elderly. A U-shaped association of changes in ABSI and mortality from all causes was detected. Compared with ABSI changes, participants with a comparatively stable ABSI with follow-up exhibited the lowest danger of death. After controlling for potential confounding factors including habits of smoking, drinking, and exercise, medical history, waist circumference, and BMI, a U-shaped positive association between ABSI changes and all-cause mortality was still observed. Further analysis clarified hazard ratios between participants‘ ABSIs, reduced or incremented by 10%, and mortality (HRs and 95% CIs per 10% ABSI change: 1.094, 1.090–1.099 1.019, 1.018–1.020, respectively). Our results emphasized the importance of simply measuring ABSI at a singular time point as well as tracking alterations in ABSI among older adults.

Elevation in ABSI is a crucial indicator of increased risk of death in metabolic disorders, such as obesity, diabetes, and CVD. Furthermore, health conditions such as peritoneal effusion and ascites can also contribute to an increase in waist circumference, which in turn increases ABSI. Previous studies have indicated that peritoneal effusion or ascites are critical complications that may arise from liver cirrhosis, renal diseases, or cancer, and have been found to be associated with accelerated mortality [38,39]. This may be another reason for the significantly greater risk observed in subjects who experienced a 1.9% increase in ABSI changes. It should be noted that there are other factors besides effusion and ascites that can lead to an increase in ABSI over time. Nevertheless, our study was constrained by the absence of data in CLHLS on the underlying causes of increased ABSI alteration, including peritoneal effusion and ascites.

A decline in ABSI over time may be linked to body mass wasting. Previous studies have demonstrated that individuals with low ABSI are at a leading risk of malnutrition, which is a known risk factor for increased mortality [40]. Our finding of a significant increase in the risk of death with a reduction in ABSI changes over time aligns with this evidence. Additionally, reduced ABSI groups showed a greater risk of death than increased ABSI groups (HRs for the two groups: 1.019; 1.094). Although our results did not provide evidence supporting the theory of the “obesity paradox” at measuring a single point for ABSI, we still found that elderly people with malnutrition or body mass wasting are more at risk than those with increased ABSI for death from all causes.

Furthermore, the U-shaped association of ABSI alteration with overall mortality was also more pronounced among individuals with a normal calf circumference, while it transformed into an L-shaped association among those with a low calf circumference. In other words, for older adults with a low calf circumference, an increment in ABSI in 3 years is associated with reduced risk of mortality. Currently, a dearth of comprehensive information exists that can help us comprehend the reasons behind the variation in the relationship with low calf circumference groups. Calf circumference is an anthropometric indicator for the older adults to assess muscle mass and malnutrition risk [41]. Several research works have claimed that fat loss predicted an adverse prognosis, independent of a patient’s body weight [42]. Attenuation of visceral adipose tissue may serve as a biomarker for both current and past nutritional status [43]. Regarding our data, older adults with low calf circumferences were at risk of malnutrition; increased ABSI may indicate that they have some visceral fat and a part of subcutaneous fat, which may confer some protective effects against mortality. Visceral fat is more easily metabolized and can be used as an energy source during periods of malnutrition. As a result, short-term increases in ABSI may serve to smooth curves among ABSI 10% increase groups. Further studies are required to shed light on the underlying mechanisms.

This study represents a significant contribution to the existing literature by providing novel insights into the utilization of ABSI in predicting risk of mortality in Chinese older adults and the potential benefits of monitoring changes in ABSI. The study’s strengths are numerous, including its thorough adjustment for relevant confounding variables, which enhances the internal validity of the findings. Our study estimates the HRs in a 10% fluctuation, filling the gap of the effect of ABSI changes on death risks. Moreover, the accurate measurement of waist circumference, height, and weight using standardized protocols further increases the credibility and reliability of the results. Overall, this study underscores the importance of monitoring changes in ABSI as a potential means of promoting health and well-being in older adults.

Despite the considerable contributions of this study, several limitations are addressed. The potential bias due to the self-reporting of coexisting medical conditions may have resulted in an incomplete accounting of all relevant information. In addition, our study only conducted a short-term 4-year follow-up, which may diminish its effects. Information about habitual dietary intake, nutritional status, and sarcopenia were not studied, which may be associated with ABSI changes and introduce bias. Finally, confounding remains a potential source of bias, even with the highly rigorous statistical techniques employed in the cohort studies. Given these limitations, future studies should strive to address these issues through improved measurement methods and the careful consideration of covariates.

In addition, according to CLHLS data gathered in the follow-up, older people may have different anthropometric measurements (such as waist circumference and BMI in calculation of ABSI) as a result of the 3-year social isolation brought on by COVID-19. Self-reported or home-measured heights, weights, or waist circumferences are likely to have been predominately obtained via telemedicine; nevertheless, they may be much less accurately acquired and gathered than those measured by a health care physician in a home visit. Some studies have presented that switching to telemedicine enabled continuous data gathering during the pandemic; however, accompanying this, the frequency of anthropometric measures sharply fell [44]. Therefore, when using the anthropometric data acquired by CLHLS or other similar databases during COVID-19 to further research ABSI and the progression of various illnesses, one should consider any potential bias from this. On the other hand, many people have experienced modest to significant changes in their lifestyles (diet and physical activities) or emotions because of the epidemic and its constraints. Some chronic diseases were shown to be much worse following high levels of social isolation, according to a Korean study [45]. We hypothesized that physical and emotional stress introduced by the social isolation during the COVID-19 pandemic might play roles in ABSI changes. Future studies that focus on ABSI changes relating to COVID-19 and the risk of adverse outcomes, especially in older populations, who are among the most vulnerable while combating COVID-19, are encouraged. 

## 5. Conclusions

Strong relationships of ABSI and its changes with mortality have been revealed from our findings in this cohort study. The mortality risk extends beyond an ABSI decrease or increase over time compared with a stable ABSI. Medical practitioners should prioritize the practical application and diligent monitoring of ABSI across various populations, with particular emphasis on the elderly, due to its convenient accessibility and utility. However, if clinical or laboratory examinations can identify individuals who may benefit from early intervention, further investigation is necessary to establish the correlation between changes in ABSI (either decreases or increases) and the onset of life-threatening conditions.

## Figures and Tables

**Table 1:** Characteristics of participants regarding ABSI tertiles at baseline.

Characteristics	Total	T1 ≤0.080	T2 0.081~0.088	T3 ≥0.089	*p*
N	3789	1262	1264	1263	
Relative body shape index (%)	−0.74 ± 15.98	−11.15 ± 15.07	−0.2 ± 11.12	9.15 ± 14.48	<0.001
Calf circumference (cm)	30.81 ± 6.49	30.70 ± 6.77	31.32 ± 6.50	30.41 ± 6.16	0.002
Body mass index (kg/m^2^)	21.81 ± 3.71	22.67 ± 3.72	22.11 ± 3.53	20.65 ± 3.60	<0.001
Waist circumference (cm)	80.98 ± 11.88	73.30 ± 11.52	82.40 ± 9.40	87.24 ± 10.10	<0.001
Age (year)	84.43 ± 10.00	83.64 ± 10.24	83.04 ± 9.61	83.61 ± 9.81	<0.001
Gender (*n*, %)					<0.001
Male	1824(48.1%)	658(17.4%)	661(17.4%)	505(13.3%)	
Female	1965(51.9%)	604(15.9%)	603(15.9%)	758(20.01%)	
Marital status (*n*, %)					<0.001
Married	1582(42.2%)	545(14.5%)	579(15.4%)	458(12.2%)	
Living alone	2168(57.8%)	708(18.9%)	668 (17.8%)	792(21.1%)	
Residence (*n*, %)					0.198
City	479(12.6%)	149(3.9%)	174(4.6%)	156(4.1%)	
Town	1313(34.7%)	422(11.1%)	428(11.3%)	463(12.2%)	
Rural	1997(52.7%)	691(18.2%)	662(17.5%)	644(17.0%)	
Drinking (*n*, %)					0.005
Yes	644(17.1%)	226(6.0%)	239(6.4%)	179(4.8%)	
No	3117(82.9%)	1023(27.2%)	1022(27.2%)	1072(28.5%)	
Smoking (*n*, %)					0.003
Yes	679(18.0%)	251(6.7%)	240(6.4%)	188 (5.0%)	
No	3094(82.0%)	1009(26.7%)	1019(27.0%)	1066(28.3%)	
Exercise (*n*, %)					0.033
Yes	1129(30.4%)	373(10.1%)	396(10.7%)	360(9.7%)	
No	2582(69.6%)	854(23.0%)	847(22.8%)	881(23.7%)	
Hypertension (*n*, %)	1287(35.9%)	408(11.4%)	467(13.0%)	412(11.5%)	0.01
Diabetes (*n*, %)	244(6.4%)	68(1.9%)	88(2.5%)	68(1.9%)	0.132
Heart disease (*n*, %)	498(14.0%)	147(4.1%)	167(4.7%)	184(5.2%)	0.061
Stroke or CVD (*n*, %)	308(8.7%)	101(2.9%)	106(3.0%)	101(2.9%)	0.923
Vital status (*n*, %)					<0.001
Dead	1342(35.4%)	449(11.9%)	385(10.2%)	508(13.4%)	
Alive	2447(64.6%)	813(21.5%)	879(23.2%)	755(19.9%)	

Notes: Continuous variables are presented as mean ± SD and were calculated using one-way ANOVA test. Categorical variables are presented as percentage and were calculated using chi-square test. Abbreviations: CVD, cardiovascular disease; T1 to T3, Tertiles 1 to 3.

**Table 2:** Associations of ABSI with mortality.

	Total (*n*)	Death (*n*)	Crude Model		Model 1		Model 2		Model 3	
			HR (95% CI)	*p*	HR (95% CI)	*p*	HR (95% CI)	*p*	HR (95% CI)	*p*
ABSI z-score	3789	1342	1.071 (1.015, 1.131)	0.013	1.397 (1.159, 1.683)	<0.001	1.223 (1.005, 1.489)	0.045	1.293 (1.046, 1.599)	0.018
ABSI tertiles										
T3	1263	508	Reference		Reference		Reference		Reference	
T2	1264	385	0.715 (0.626, 0.816)	<0.001	0.906 (0.777, 1.056)	0.206	0.916 (0.783, 1.071)	0.270	0.874 (0.738, 1.053)	0.118
T1	1262	449	0.847 (0.746, 0.96)	0.010	0.992 (0.809, 1.218)	0.942	1.003 (0.813, 1.237)	0.978	0.925 (0.735, 1.164)	0.505

Mode 1: adjusted age, sex, BMI, waist circumference. Model 2: adjusted for covariates in Model 1 and additionally adjusted for marital status, residence, smoking, drinking, exercise. Model 3: adjusted for covariates in Model 2 and additionally adjusted for hypertension, diabetes, heart disease, and stroke or CVD. Abbreviations: T1 to T3, Tertiles 1 to 3; HR, hazard ratio; 95% CI, 95% confidence intervals; *p*, *p*-value.

**Table 3:** Hazard ratios of all-cause mortality with 10% ABSI change effect.

	Per 10% Increase in ABSI		Per 10% Reduction in ABSI	
	HRs	95% CI	HRs	95% CI
Crude model	1.062	1.059–1.065	1.096	1.091–1.100
Model 1	1.026	1.025–1.027	1.044	1.042–1.045
Model 2	1.027	1.026–1.028	1.038	1.037–1.040
Model 3	1.043	1.041–1.045	1.054	1.052–1.057
Model 4	1.019	1.018–1.020	1.094	1.090–1.099

Model 1: age, sex. Model 2: Model 1 additionally adjusted for marital status, residence, smoking, drinking, and physical exercise. Model 3: Model 2 additionally adjusted for hypertension, diabetes, heart disease, stroke, and cardiovascular disease. Model 4: Model 3 additionally adjusted for calf circumference, BMI, waist circumference, and ABSI at baseline. Abbreviations: HR, hazard ratio; 95% CI, 95% confidence intervals.

## Data Availability

The original data used in this paper were derived from the Chinese Longitudinal Healthy Longevity Survey (CLHLS). The data are available from the website: https://opendata.pku.edu.cn/dataset.xhtml?persistentId=doi:10.18170/DVN/WBO7L (accessed on 3 April 2020). Processed data described in the manuscript, code book, and analytic code will be made available upon request pending an approval from the corresponding author.
